# Plasma Proteomics Unveil Novel Immune Signatures and Biomarkers upon SARS-CoV-2 Infection

**DOI:** 10.3390/ijms24076276

**Published:** 2023-03-27

**Authors:** Víctor Urbiola-Salvador, Suiane Lima de Souza, Peter Grešner, Talha Qureshi, Zhi Chen

**Affiliations:** 1 Intercollegiate Faculty of Biotechnology of University of Gdańsk and Medical University of Gdańsk, University of Gdańsk, 80-307 Gdańsk, Pomerania, Poland; 2 Faculty of Biochemistry and Molecular Medicine, University of Oulu, 90220 Oulu, North Ostrobothnia, Finland; 3 Department of Translational Oncology, Intercollegiate Faculty of Biotechnology of University of Gdańsk and Medical University of Gdańsk, Medical University of Gdańsk, 80-211 Gdańsk, Pomerania, Poland

**Keywords:** SARS-CoV-2, plasma proteomics, COVID-19, immune signature, biomarker

## Abstract

Several elements have an impact on COVID-19, including comorbidities, age and sex. To determine the protein profile changes in peripheral blood caused by a SARS-CoV-2 infection, a proximity extension assay was used to quantify 1387 proteins in plasma samples among 28 Finnish patients with COVID-19 with and without comorbidities and their controls. Key immune signatures, including CD4 and CD28, were changed in patients with comorbidities. Importantly, several unreported elevated proteins in patients with COVID-19, such as RBP2 and BST2, which show anti-microbial activity, along with proteins involved in extracellular matrix remodeling, including MATN2 and COL6A3, were identified. RNF41 was downregulated in patients compared to healthy controls. Our study demonstrates that SARS-CoV-2 infection causes distinct plasma protein changes in the presence of comorbidities despite the interpatient heterogeneity, and several novel potential biomarkers associated with a SARS-CoV-2 infection alone and in the presence of comorbidities were identified. Protein changes linked to the generation of SARS-CoV-2-specific antibodies, long-term effects and potential association with post-COVID-19 condition were revealed. Further study to characterize the identified plasma protein changes from larger cohorts with more diverse ethnicities of patients with COVID-19 combined with functional studies will facilitate the identification of novel diagnostic, prognostic biomarkers and potential therapeutic targets for patients with COVID-19.

## 1. Introduction

The ongoing COVID-19 pandemic, which is caused by SARS-CoV-2, is the most significant catastrophe that humanity has faced in the 21st century. With several widespread variants, more than 6 million COVID-19-related deaths, and over 600 million cases [1], COVID-19 is ranked as one of the deadliest pandemics in recorded human history [2]. Despite all of the advancements in medical sciences, preventive measures, and efficient vaccination programs, the COVID-19 pandemic still persists, and thousands of COVID-19-related deaths are reported every day [1].

Regarding the immune reaction upon SARS-CoV-2 infection, a large amount of variability is observed among the human population, and current studies show that immunopathology is largely responsible for COVID-19 pathogenesis and its related mortality [3,4,5]. The release of large quantities of pro-inflammatory cytokines, which is known as a cytokine storm, is considered as an underlying reason for the hyperactive immune response against SARS-CoV-2 infection and is correlated with COVID-19 disease severity [6]. The ample production of cytokines attracts immune cells to the site of infection and causes tissue damage that may lead to pneumonia, lung injury and multi-organ failure, which are complications commonly seen in critical and deceased patients with COVID-19 [7].

Although vaccines have shown a promising outcome to curb the spread of COVID-19 infection [8], the rate of mutation in the SARS-CoV-2 single-stranded RNA-based genome will likely result in a greater landscape of variants. The outright eradication of COVID-19 seems improbable in the near future [9,10,11]. Importantly, despite that the majority of patients with COVID-19 do not develop severe symptoms that require intensive care unit (ICU) admission, several patients contract post-COVID-19 syndrome characterized by multi-organ symptoms that persist for months after acute COVID-19, independently of the disease severity [12]. A remarkable number of studies have been published on COVID-19 since the inception of the pandemic. Nevertheless, our current knowledge of the immunological changes upon SARS-CoV-2 infection is still incomplete. There is a pressing need to broaden our understanding of the immune dynamics that occur upon this viral infection as well as the long-term effects of post-COVID-19 syndrome.

COVID-19 progression and severity are determined by age, sex, ethnicity, comorbidities and some risk genetic mutations carried by patients [13,14,15,16], which make it rather difficult to pinpoint the immune signatures that could be used to anticipate a hyperactive immune response. Importantly, comorbidities, such as cancer, diabetes and cardiovascular and neurological diseases, are reported to be associated with higher severity and increased risk of death in patients with COVID-19 [17,18,19,20]. Albeit interpatient heterogeneity of medical conditions, there could be key signature proteins that may serve to determine the underlying immune and physiological responses in SARS-CoV-2-infected patients with comorbidities. Recent studies show that around 20% of people infected by SARS-CoV-2 may continue to develop symptoms diagnosed as post-COVID-19 condition (also known as long COVID), a condition about which there still remains limited information [12,21].

In this study, we investigated the plasma proteomic profiles of patients with COVID-19 from a Finnish biobank by proximity extension assay (PEA) [22] with reference to age and sex-matched disease controls (DC) and healthy controls (HC) collected in Finnish Clinical Biobank, Tampere, to understand how and which proteins are diverted from their normal expression patterns that may lead to disruption in immune features and cellular functionality. Among the identified protein changes, many key players in immune regulation and inflammation were upregulated, especially in patients with comorbidities. Moreover, several protein changes were associated with the generation of specific SARS-CoV-2 antibodies and time after infection that reflects the long-term effects of post-COVID-19 condition.

Our analyses provide deeper insights into the plasma proteomic changes caused by a SARS-CoV-2 infection that modulate the immunological and physiological response in the Finnish population. Several novel plasma proteins involved in innate and adaptive immunity, T cell activation/cycling, and extracellular matrix (EMC) remodeling were associated with SARS-CoV-2 infection. Given the cohort size of this study was relatively small and samples were from a single center in Finland, the further characterization of these plasma protein changes with a larger cohort consisting of more diverse ethnicity may lead to the identification of novel diagnostic and prognostic biomarkers as well as potential therapeutic targets for patients with COVID-19.

## 2. Results

### 2.1. SARS-CoV-2 Infection in Patients with Comorbidities Causes Plasma Protein Changes with Enhanced Soluble CD4 and Associated Proteins

**Figure 1:**
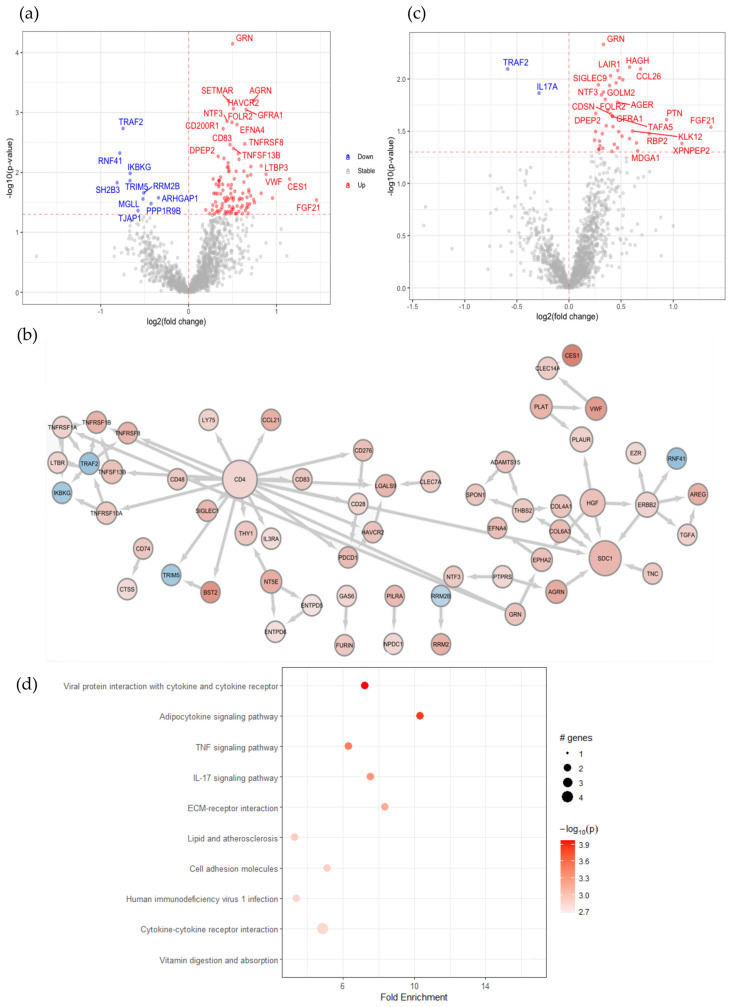
SARS-CoV-2 infection in patients with comorbidities causes plasma protein changes with enhanced soluble CD4 and associated proteins. (**a**) Volcano plot of statistical significance against fold-change of proteins between SARS-CoV-2 virus-infected patients with comorbidities and healthy controls. Colored dots indicate statistically differentially expressed proteins (DEPs); (**b**) protein–protein interaction network of DEPs between SARS-CoV-2 virus-infected patients with comorbidities and healthy controls with the organic layout from the STRING database query with a 0.7 confidence cut-off. The size of nodes indicates the degree of connectivity of the nodes; (**c**) volcano plot of statistical significance against fold-change of proteins between SARS-CoV-2 virus-infected patients with comorbidities and paired disease controls. Dots indicate statistical DEPs; (**d**) dot plot of KEGG pathway enrichment combined with STRING protein–protein interaction network analysis from DEPs between patients and disease controls. (**a**–**c**) The red and blue dots represent upregulation and downregulation in patients, respectively.

To determine the changes in the protein profiles in peripheral blood caused by SARS-CoV-2 infection, we performed plasma protein analysis by using proximity extension assay technology. Out of the total 1472 proteins from the entire Explore panels, after removing repetitions in the panels and the filtration of proteins with low detection rates among the samples, 1387 proteins were quantified in our samples. The mean intra-assay coefficient of variation (CV) was 12.25% whereas the inter-assay CV was not available as all the samples were included in the same sample plate. In our cohort, 19 out of the 28 patients with COVID-19 have comorbidities in addition to SARS-CoV-2 infection, such as type 2 diabetes mellitus, asthma, cancer, multiple sclerosis and rheumatoid arthritis. Firstly, we compared the plasma protein changes between the samples from the SARS-CoV-2 virus-infected patients with comorbidities versus their HCs. Among all of the 1387 quantified proteins, the expression of 116 proteins was significantly changed (Figure 1a). Among these differently expressed proteins (DEP), 106 were upregulated in patients with comorbidities, such as ACE2, FOLR2 and AGRN, and 10 were downregulated, such as RNF41 and TRAF2. The membrane-bound ACE2 is essential to facilitate the entry of SARS-CoV-2 [23]. Importantly, ACE2 was among the elevated proteins detected in this group of patients with COVID-19 (Figure 1a and Figure S1a and Table S1). A recent study also indicates that the interaction of the SARS-CoV-2 spike protein with soluble ACE2 (sACE2) or with a complex of sACE2 and vasopressin leads to the receptor-mediated endocytosis of the virus [24]. Next, in order to further identify their expression patterns, we clustered the 116 identified DEPs (Figure S1a). Hierarchical clustering clearly distinguishes patients with comorbidities with more upregulated and less downregulated proteins observed versus HCs. Notably, the helper T cell surface marker CD4, the co-stimulatory molecule of T cells CD28 and the B-cell activation protein CD83 were found in this group, indicating the active involvement of immune cells in response to a SARS-CoV-2 infection. To distinguish which of the protein changes were due to an altered secretion from a certain type of cells and which were a result of destructed tissues or cells due to the viral infection, from the 116 DEPs, 30 proteins were identified in the human blood secretome, such as FOLR2, GRN, EFNA4, TNFSF13B, CCL26, CCL21, PILRA, VWF, LTBP3, LILRA5 and TGFA (Table S2).

To understand the highly complex interaction patterns of the DEPs, we constructed a protein–protein interaction network using the STRING *Homo Sapiens* database. CD4 is highly expressed on the surface of helper T cells [25]. Previously, the soluble form of CD4 (sCD4) has been reported to be detected in patient serum with an HIV infection, and the sCD4-to-CD4 lymphocyte ratio increases with disease severity [26]. Here, we observed an increased level of CD4 in the SARS-CoV-2 infected plasma samples compared to plasma from healthy donors. The network analysis showed the association of CD4 with multiple identified DEPs, including CD28, CD48, CD83 and PDCD1 (also named PD1), which were also elevated in patients’ plasma compared to their HCs (Figure 1b), suggesting the potential damage of helper T cells in response to a SARS-CoV-2 infection. Furthermore, a subgroup of TNF and TNF receptor superfamily members, including TNFSF13B, TNFRSF8, TNFRSF6B, TNFRSF1B, TNFRSF1A and TNFRSF10A was elevated in patients’ plasma and formed a small network (Figure 1b). The elevated level of these proteins in SARS-CoV-2-infected plasma suggests the active regulation of this pathway in blood cells in response to this viral infection. Moreover, pathway enrichment analysis showed the upregulation of proteins related to extracellular matrix (ECM)–receptor interaction that is involved in ECM remodeling, tissue damage and repair (Figure S1b). Viral infection can activate innate and adaptive immune responses, in which the NF-κB signaling pathway plays an important role; on the other hand, viruses may suppress NF-κB pathway activation to dampen the host immune responses [27,28]. Not surprisingly, pathway analysis of the DEPs in a comparison of 19 patients with comorbidities versus HCs showed the enriched positive regulation of the I-kappaB kinase/NF-κB signaling pathway and the positive regulation of viral protein interaction with cytokine and cytokine receptors (Figure S1b). Collectively, SARS-CoV-2 infection clearly causes changes of proteins in peripheral blood that are associated with immune responses and especially might induce T cell death as indicated by the enhanced soluble CD4 and its associated proteins.

The clustering analysis showed that some of the protein expression changes identified in patients infected with SARS-CoV-2 were also observed in their age and sex-matched DCs, such as proteins in cluster 3 in Figure S2a, whereas out of the 116 DEPs in patients with comorbidities, 16 were also changed when we compared their DCs with HCs, indicating changes in these plasma proteins may be caused by their comorbidities (Figure S2b).

To dissect what proteins were indeed affected by COVID-19 but not due to their comorbidities, we compared the group with COVID-19 and comorbidities to their matched DCs but without SARS-CoV-2 infection. From the 44 DEPs, all of the proteins were upregulated in patients with COVID-19 compared with their DCs, except TRAF2 and IL17A (Figure 1c, Table S3).

KEGG pathway enrichment combined with STRING protein–protein interaction network analysis of the DEPs revealed a reduced IL-17 signaling pathway (Figure 1d). On the other hand, among the 42 elevated proteins in patients with comorbidities compared with their DCs, 28 plasma proteins, including CD28 were found also elevated when compared with their HC, indicating that the elevated plasma level of these 28 proteins is most likely caused by COVID-19 infection but not the comorbidities. FGF21 and NTF3, associated with the MAPK-signaling pathway, TNFRSF1B, and CCL26 associated viral protein interaction with cytokine and cytokine receptor pathway were upregulated in plasma proteins from patients with COVID-19 with comorbidities compared to their DCs, indicating that COVID-19 caused changes of these pathways. Collectively, these results demonstrate the shared plasma protein signatures in patients with COVID-19 with comorbidities, despite the heterogeneity of their medical conditions.

### 2.2. Reduced RNF41 in Plasma Is Associated with a SARS-CoV-2 Infection

**Figure 2:**
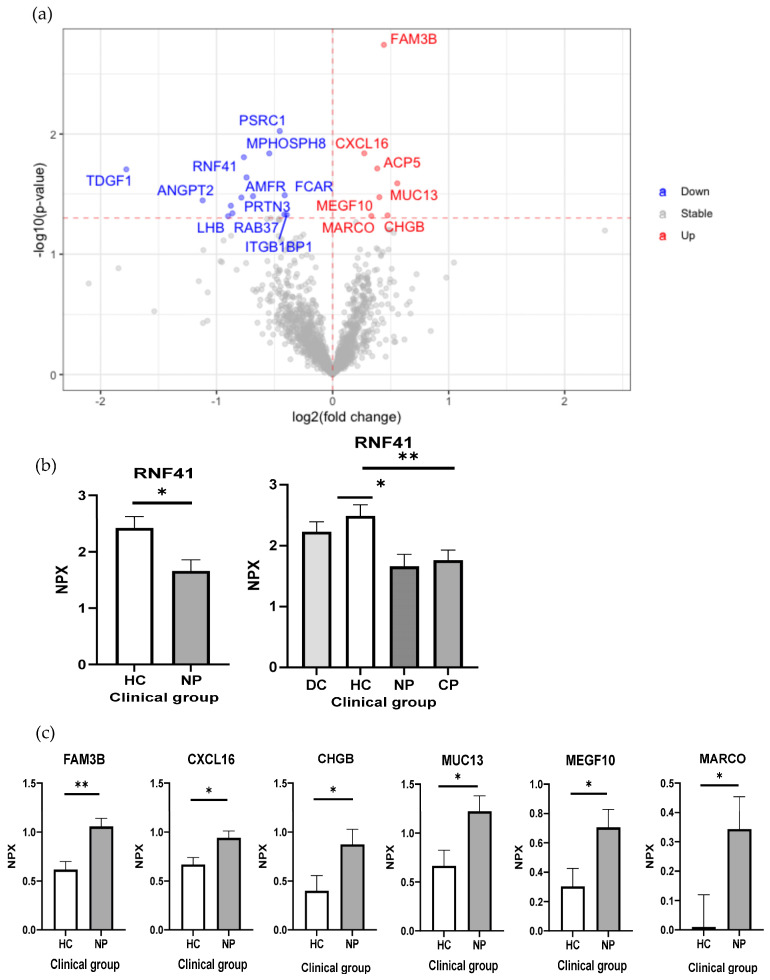
Reduced plasmas level of RNF41 is associated with SARS-CoV-2 infection. (**a**) Volcano plot of statistical significance against fold-change of proteins between patients without comorbidities and healthy controls. The red (upregulated in patients without comorbidities) and blue (downregulated in patients without comorbidities) dots indicate statistical DEPs; (**b**) bar plots with normalized protein expression of RNF41 among different clinical groups; (**c**) bar plots with normalized protein expression of FAM3B, CXCL16, CHGB, MUC13, MEGF10 and MARCO in patients without comorbidities and their respective healthy controls. * and ** indicate statistically significant with an adjusted *p*-value < 0.05 and <0.01, respectively. CP, comorbidities patient; DC, disease control; HC; healthy control; NP, non-comorbidities patient; NPX, normalized protein expression.

Next, we performed one more analysis to compare the patient samples without chronic diseases to their healthy controls to define the proteins related to the SARS-CoV-2 infection itself. From this analysis, 21 DEPs were found, 7 proteins were detected higher in patients, and 14 proteins were higher in the HCs (Figure 2a, Table S4). It is noteworthy that this comparison resulted in a lower number of DEPs. RNF41, an E3 ubiquitin–protein ligase, is among the 14 proteins with a higher level of HCs than that of patients with COVID-19. Additionally, it is the only protein with a reduced level in patients with COVID-19 with and without comorbidities compared with their HCs (Figure 1a and Figure 2a,b). RNF41 is involved in type 1 cytokine receptor signaling, regulating JAK2-associated cytokine receptor surface by degradation and ectodomain shedding [29]. RNF41, as in the other RNFs, can inhibit antiviral responses; nevertheless, with the regulation of IFN signaling, it is still unclear [30]. RNF41 expression is with low tissue specificity and is mainly involved in the B-cell immune response. It has been reported that the SARS-CoV-2 NSP15 protein targets RNF41 [31], and this interaction may be involved in antagonizing the induction of IFN-I [32]_._ In murine dendritic cells, RNF41 negatively regulates the cross-presentation of dead cell-derived antigens by the ubiquitination of CLEC9A [33]. We observed a reduced plasma level of RNF41 in patients with COVID-19, suggesting a potential function of RNF41 in SARS-CoV-2 infection. Therefore, the analysis of plasma proteomics revealed novel potential diagnostic biomarkers for COVID-19.

A group of plasma proteins was found to be elevated in patients without comorbidities compared with their HCs, such as FAM3B, CXCL16, CHGB, MUC13, MEGF10 and MARCO (Figure 2a,c).

### 2.3. Characterization of Early and Long-Term Plasma Protein Responses Associated with SARS-CoV-2 Infection

**Figure 3:**
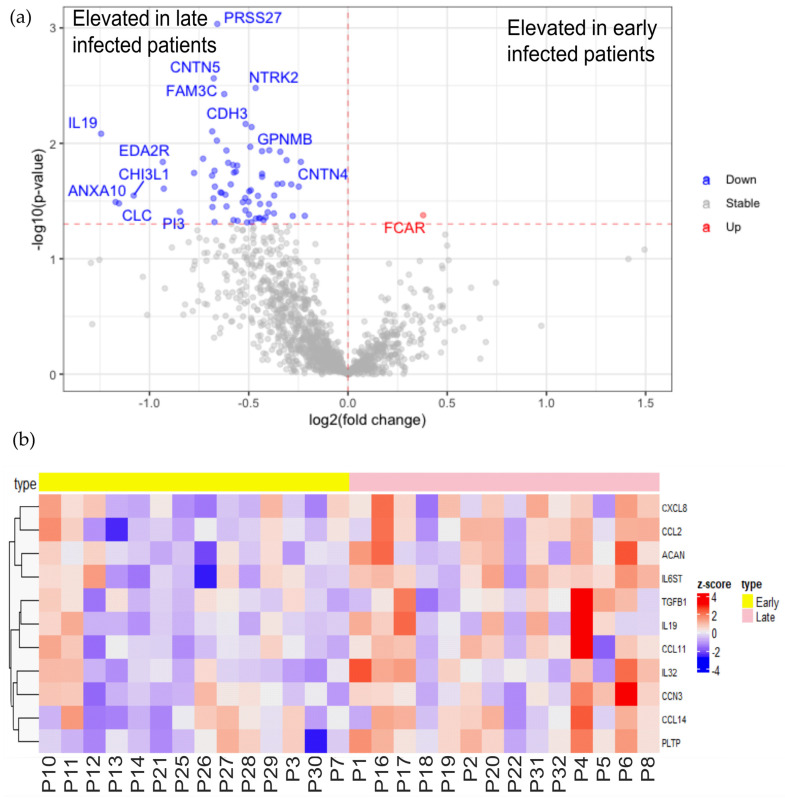
Characterization of long-term plasma protein responses associated with SARS-CoV-2 infection. (**a**) Volcano plot of statistical significance against fold-change of proteins between patients with plasma samples collected less than 3 months after infection (early) and collected more than 3 months after infection (late). Red (upregulated in patients with early collection) and blue (upregulated in patients with late collected plasma) dots indicate statistically DEPs; (**b**) heatmap of selected statistical DEPs between patients with plasma collected less than 3 months after infection (early) and collected more than 3 months after infection (late) with z-score by row normalization and distributed by hierarchical clustering.

To characterize the plasma protein response to early and late SARS-CoV-2 infection, we compared the 14 plasma samples collected within 3 months after infection with the 14 samples collected after 3 months of infection. As shown in Figure 3a, the longer period after infection caused more elevated plasma proteins than early infection. Only the receptor for the Fc region of IgA (FCAR) was significantly elevated in early infected patients. FCAR interacts with IgA-opsonized targets and triggers several immunologic defense processes. However, no association between its plasma level with COVID-19 has been reported yet. Sixty-seven plasma protein levels were higher in patients infected longer than 3 months compared to recently infected patients, including a group of cytokines and chemokines, such as IL6ST, TGFB1, IL32, IL19, CXCL8, CCL2, CCL11 and CCL14 (Figure 3, Table S5). These elevated cytokines indicate the pathologic inflammation that may drive post-COVID-19 condition [12]. Among the elevated chemokines, the eosinophil-attracting chemokine CCL11 was previously found elevated in late post-COVID-19 syndrome patients which may be associated with cognitive symptoms [34]. Meanwhile, CCN3 and PLTP were upregulated in patients with longer periods of infection which are involved in the negative regulation of inflammation [35,36]. They may attenuate COVID-19 pro-inflammatory conditions in order to re-establish homeostasis. Moreover, high plasma levels of aggrecan (ACAN), the main component of cartilaginous ECM, may indicate the degradation of cartilaginous tissues after COVID-19 pro-inflammatory conditions. In fact, aged joints and arthritis may be associated with post-COVID-19 condition [37]. Taken together, the characterization of early and long-term effects of plasma protein responses to SARS-CoV-2 infections resulted in the identification of novel potential biomarkers of post-COVID-19 condition.

### 2.4. Plasma Protein Changes Associated with SARS-CoV-2 Antibody Generation

**Figure 4:**
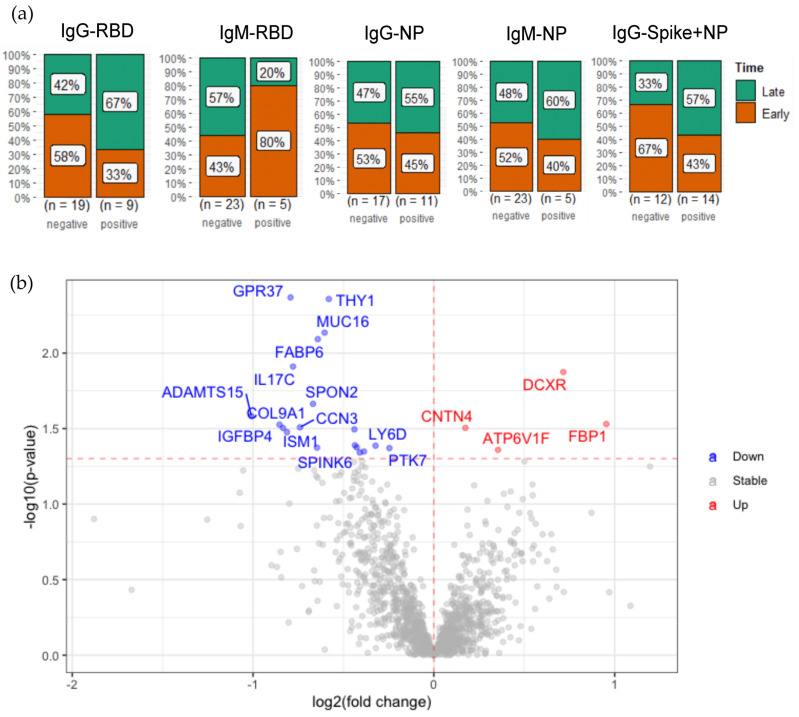
Plasma protein changes associated with SARS-CoV-2 antibody generation. (**a**) Bar plots represent the percentage of patients with positive and negative antibody generation for different SARS-CoV-2 antigens from patients with early-collected plasma (<3 months) and late-collected plasma (>3 months); (**b**) volcano plot of statistical significance against fold-change of proteins between patients with positive antibody generation and patients with negative antibody generation. The red (upregulated in patients with positive antibody generation) and blue (downregulated in patients with positive antibody generation) dots statistically indicate DEPs.

To investigate whether the plasma protein changes are associated with antibody generation, we detected SARS-CoV-2 IgM and IgG antibodies against different antigens, including spike receptor-binding domain (RBD), and nucleocapsid protein (NP). For IgG, spike combined with NP was also measured. As a result, 12 out of 28 (42.8%) of the patients infected with SARS-CoV-2 did not generate any detectable antibodies against any of the antigens (Table 1). Among the patients infected with SARS-CoV-2, no association of antibody generation with comorbidities was found. From those IgM antibody-positive patients, 80% of RBD-positive patients had a SARS-CoV-2 infection less than 3 months before the antibody measurement, whereas 67% of RBD IgG-positive patients had an infection for more than 3 months (Figure 4a). The detected IgM and IgG antibodies against SARS-CoV-2 RBD follow the regular pattern of antibody generation after a viral infection. However, the pattern in antibody detection against NP antigens is less clear (Figure 4a), suggesting that RBD is more suitable than NP as an antigen for the specific testing of a SARS-CoV-2 infection.

To identify which protein changes may be associated with the generation of antibodies in response to this viral infection, we compared the detected plasma proteins from patients with and without detectable anti-SARS-CoV-2 IgM or IgG antibodies. Among the 12 patients who did not generate detectable antibodies, we observed a significantly elevated level of IL17C and a protein in the PPAR signaling pathway, namely, FABP6, compared to patients with COVID-19 with detectable antibodies (Figure 4b, Table S6). Moreover, SPON2, involved in innate immune responses [38] was increased in these antibody-negative patients. ATP6V1 was upregulated in patients with antibody generation. This protein acidifies cellular compartments as well as the extracellular environment, which was previously found upregulated in SARS-CoV-2-infected cells [39]. Interestingly, it has been reported that an acidic pH environment facilitates SARS-CoV-2 infection [40]. Taken together, the comparison of the samples from the SARS-CoV-2 antibody-negative and -positive patients revealed plasma protein changes associated with SARS-CoV-2 antibody generation, suggesting the potential roles of these proteins in SARS-CoV-2 immune responses.

### 2.5. Identification of Key Immune Signatures and Novel Protein Changes Caused by a SARS-CoV-2 Infection

**Figure 5:**
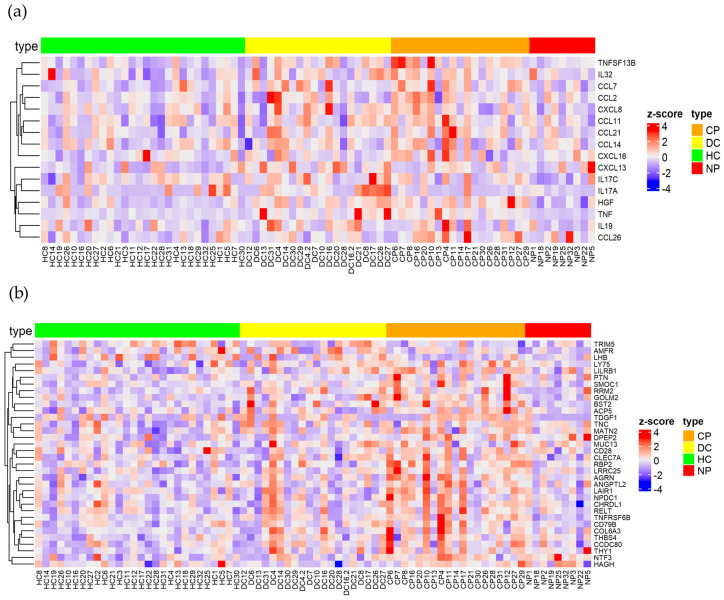
Identification of key immune signatures and novel proteins after SARS-CoV-2 infection. (**a**) Heatmap of differentially expressed cytokines and (**b**) novel protein changes among different clinical groups (patients with and without comorbidities, disease controls, and healthy controls) with z-score by row normalization and distributed by hierarchical clustering.

In this study, 220 DEPs were identified when accounting for all of the comparisons, among which, 59 proteins were identified in the human blood secretome according to HPA (Table S2). We revealed the upregulation of several chemokines and cytokines, including CCL26, HGF and TNFSF13B/BAFF, especially in patients with comorbidities, which promote inflammation, innate immune responses as well as T/B-cell-driven immune responses (Figure 5a). Interestingly, pleiotrophin (PTN), a secreted growth factor that induces the stimulation of the expression of inflammatory cytokines was upregulated in patients with comorbidities compared to their DCs (Figure 5b). This is consistent with the previous findings in which PTN was elevated at the mRNA level in the upper airway of patients with COVID-19 as well as patients with COVID-19 over the age of 60 [41,42]. Another growth factor upregulated in this group of patients was FGF21, which is linked to mitochondrial dysfunction in peripheral blood mononuclear cells (PBMCs) which drives a systemic immune response in COVID-19 pathogenesis [43]. Moreover, the patients with comorbidities showed the upregulation of well-established COVID-19-related inflammatory markers, such as AREG, an IL-18-induced cytokine, which is involved in restoring tissue integrity [44,45,46]. Simultaneously, the upregulation of LRRC25 (Figure 5b), which is a potent negative regulator of NF-κB signaling and inflammation [47], may counteract the hyper-inflammation state in patients with COVID-19. Another possible function of LRRC25 after a SARS-CoV-2 infection is the downregulation of RLR-mediated type I interferon (IFN) signaling [48]. The elevated plasma LRRC25 was detected in patients with comorbidities compared with HCs as well as with DCs, suggesting that the elevated plasma LRRC25 is caused by SARS-CoV-2 infection but not by the comorbidities. Notably, the elevated plasma level of LRRC25 detected in patients with COVID-19 has not previously been reported.

Several proteins related to innate immune cell activation are upregulated in patients with COVID-19, such as FOLR2, anti-inflammatory macrophage markers [49,50], and CCL26. Notably, elevated levels of these two proteins were detected in patients with COVID-19 with and without comorbidities (Figure S3). CCL26 is highly expressed in vascular endothelium, fibroblasts, epithelial, and blood endothelial cells [51,52]. It is a chemoattractant to recruit inflammatory cells, especially eosinophils and mast cells in allergic reactions and other immune diseases. It may also block the recruitment of Th1 and monocytes via CCR1, -2, and -5 [53]. CCL26 regulates the proportion of CD4+CD25+FOXP3+ Tregs and the production of inflammatory factors in PBMCs following acute ischemic stroke via the STAT5 pathway [54]. Studies have shown that SARS-CoV-2 ORF7a activates the NF-KB pro-inflammatory release of CCL26 among other proinflammatory cytokines [55]. Previously, CCL26 was found to be upregulated in patients with COVID-19 vs. HCs in plasma and correlates with disease severity [56,57].

Apart from the elevated plasma proteins, we also identified several downregulated proteins after a SARS-CoV-2 infection, among which, besides the abovementioned RNF41, surprisingly, the inflammatory cytokine IL-17A was found to be increased in DCs but reduced in patients with COVID-19 with comorbidities compared to their DCs (Figure 1c, Figure 5a and Figure S3). The elevated plasma IL-17A detected in DCs, especially in patients with multiple sclerosis, arthropathic psoriasis and diverticular disease of the large intestine supports the previous findings showing its association with autoimmune inflammation [58]. However, we did not observe a significant change in plasma IL-17A in response to SARS-CoV-2 infection, regardless of the presence or absence of comorbidities. This suggests that there is no clear association between IL-17A and this viral infection in the samples of patients with COVID-19 that we detected.

Furthermore, we identified several plasma proteins, including RBP2, MATN2, THY1, SMOC1, CHRDL1, NPDC1, GOLM2 and RELT, which were not previously associated with COVID-19 (Figure 5b). RBP2 has a function in the absorption of dietary retinoids. Human RBP2 bound all-trans-retinol and all-trans-retinaldehyde but not all-trans-retinoic acid. The RBP2 protein is highly expressed in the intestine (Figure S4) and plays a central role in maintaining intestinal innate immunity: Dendritic cells use all-trans-retinoic acid to promote intestine-specific immune responses, including Foxp3+ Treg conversion, lymphocyte gut-homing molecule expression, and IgA production. RBP2 is required for CD103+ DCs with the ability to generate gut tropic T cells [59]. We found elevated plasma RBP2 in patients with COVID-19 with comorbidities compared to the HCs as well as to their DCs (Figure 5b and Figure S3), but no significant changes were observed in patients without comorbidities vs. HCs. This indicates that the elevated RBP2 is the synergistic effect of comorbidities and SARS-CoV-2 infection. Other proteins, including SMOC1 and GOLM2, were also found to be elevated in SARS-CoV-2 patients with comorbidities. SMOC1 plays essential roles in both eye and limb development, whereas GOLM2’s function is not well characterized but was associated with breast cancer and glioblastoma [60,61]. However, these two proteins have not been associated with viral infection or immune response, and further studies should be performed to assess their relations with the pathophysiology of COVID-19.

Taken together, plasma proteomics analysis in patients with COVID-19 revealed protein changes with immunological signatures. In addition to SARS-CoV-2 infection, comorbidities cause plasma protein changes. Several novel potential biomarkers associated with SARS-CoV-2 infection in plasma were identified. Further functional characterization of these proteins in COVID-19 will lead to a better understanding of the immune response in a SARS-CoV-2 infection and may facilitate the development of novel therapeutic targets, diagnosis and prognosis of COVID-19.

## 3. Discussion

In the present study, we applied an antibody-based proteomic technology, a proximity extension assay to detect 1463 proteins, including inflammation, oncology, neurology and cardiometabolic panels from just a few microliters of plasma samples from patients with COVID-19. The technology is based on target-specific antibodies conjugated with unique complementary DNA. The antibody pairs targeting one protein bind to the target and a barcoded DNA duplex is formed, which is amplified by next-generation sequencing (NGS) [22]. Due to its high sensitivity and low sample volume requirement, PEA technology has been applied to the discovery and monitoring of biomarkers as well as to the diagnosis and prognosis of several diseases, such as infection, inflammation, cardiovascular diseases, neurological diseases and cancer [62,63,64,65,66]. However, like all other antibody-based approaches, this technology is limited by the availability and specificity of antibodies, and, more importantly, the number of proteins is preselected. Nevertheless, the application of PEA plasma proteomics enabled us to identify 34 novel potential biomarkers for SARS-CoV-2 infection, such as RNF41, FOLR2, RBP2, PTN, LILRA5 and CLEC7A, among others.

In the present study, immune signatures including both innate and adaptive immunity were identified in the plasma samples of patients with COVID-19. Several proteins with a function in innate immune responses were found to be upregulated in patients with COVID-19. In addition to CCL26 and FOLR2, LILRA5, which is an orphan receptor that stimulates cytokine production in monocytes [67], was also upregulated. To the best of our knowledge, this is the first time that LILRA5 has been identified as being upregulated in the serum of patients with COVID-19 as a novel potential biomarker, which was only previously found to be upregulated in the kidneys of patients with COVID-19 at the mRNA level [68]. Moreover, patients with comorbidities showed the upregulation of Dectin-1 (CLEC7A), a pattern-recognition receptor (PRR) that stimulates NFAT activation in DCs and macrophages [69] and may participate in cross-communication with TLRs during S protein and DAMP identification and stimulation [70]. Simultaneously, some immune suppressors are upregulated that potentially dampen the immune response, such as VSIG4 inhibiting macrophages and T cell cytotoxicity [71,72,73], PILRA that was previously found to be upregulated in monocytes from severe-stage patients with COVID-19 [74], and SIGLEC9 that potentially inhibits NK cells and neutrophils [75,76]. As expected, SARS-CoV-2 infection promotes the expression of genes directly related to antiviral activity such as CCL26, GRN and BST2. In fact, tetherin (BST2) is a transmembrane protein with antiviral activity by tethering nascent virions in the plasma membrane, which can be retained or mobilized for endocytic internalization and subsequent ubiquitin-based degradation [77].

SARS-CoV-2 infection promotes T cell activation and cycling [78]. In fact, patients with COVID-19 presented the upregulation of T cell markers, such as CD4, CCL21, CD48 and TNFRSF1B/TNFR2. Interestingly, our dataset revealed several upregulated plasma proteins that have not previously been reported to be associated with SARS-CoV-2 infection, such as RELT and THY1. RELT is a member of the TNF receptor superfamily and was shown to activate the NF-kB signaling pathway and is capable of stimulating T cell proliferation in the presence of CD3 signaling that may promote T immune cell responses against SARS-CoV-2 [79]. Although the function of soluble THY1 is unclear, the membrane-bound form was reported to promote neutrophil and monocyte adhesion to endothelial cells and Th17 activation to enhance the host defense against pathogens, SARS-CoV-2 in this case [80,81]. In concordance, several markers of APC activation were also found to be upregulated such as CD83 and CD74 involved in MHCII antigen presentation [82]. Simultaneously, inhibitory markers were upregulated in patients with COVID-19 that potentially counterbalance the hyperactivation of the immune response, such as NT5E/CD73, PD1 and TNFRSF8/CD30. Another novel elevated protein in this group is LAIR1, which downregulates IL-2 and IFN-γ expression in CD4+ T cells as well as IgG production, IL8, IL10 and TNF secretion in B cells [83,84]. Moreover, the upregulation of LGALS9 and its receptor HAVCR2 (TIM3) in this group of patients suggests the exhaustion of CD4+ T cells [85].

Apart from immune-related proteins, our dataset revealed the upregulation of proteins involved in metabolic processes, such as leukotrienes synthesis (DPEP2 and LTA4H), histamine degradation (HNMT), retinoic acid synthesis (RBP2) and fatty acid metabolism (FABP1). In addition, proteins related to neuronal damage were found to be upregulated, such as AGRN, NTF3, NPDC1 and CHRDL1/CHL1. NPDC1 plays a role in the control of neural cell proliferation and differentiation [86] whereas CHRDL1 is secreted by astrocytes and plays essential roles in many developmental processes, including neurogenesis, vascular development, angiogenesis and osteogenesis [87]. For the first time, we reported elevated plasma NPDC1 and CHRDL1 in patients with COVID-19 with comorbidities. Their roles in the pathogenesis of COVID-19 need to be further characterized. Moreover, extracellular matrix proteins were found to be upregulated in patients with COVID-19 that indicates the potential tissue damage and remodeling due to a SARS-CoV-2 infection such as the pro-inflammatory TNC [88], COL6A3, MATN2 and THBS4/TSP4, which are involved in tissue regeneration and wound healing [89], not previously found in patients with COVID-19. In agreement with previous findings, the Von Willebrand factor (VWF) was upregulated in patients with COVID-19 which can contribute to their hypercoagulable state and increased venous thromboembolism rate [90,91].

Several studies have demonstrated that the underlying medical conditions may increase the risk of infection with SARS-CoV-2 or the severity of COVID-19 [17,18,19,20]. Importantly, the majority of the population with SARS-CoV-2 infection does not develop severe symptoms that require admission to ICUs; therefore, our cohort nicely represents the overall situation in the population [1]. However, recently, more attention has been drawn to the fact that a considerable amount of people infected by SARS-CoV-2 may develop long COVID, a condition of which we currently have very limited knowledge. In the present study, the application of the PEA, an antibody-based proteomic strategy, resulted in the quantification of over 1000 plasma protein changes in patients with COVID-19 mostly with mild symptoms. Further analysis unveiled immunological signatures and several novel protein changes associated with SARS-CoV-2 infection, their underlying diseases, as well as antibody generation. In fact, our data demonstrated that patients with COVID-19 with comorbidities shared plasma protein signatures that reflect their underlying immune and physiological responses, despite the heterogeneity of medical conditions. Furthermore, the characterization of long-term plasma protein responses resulted in the identification of novel potential biomarkers for post-COVID-19 condition. It must be noted that the relatively small number of patients from our Finnish cohort from a single center limits the statistical power of this study and its applicability to the overall population. However, the robust study design and statistical analysis as well as the consistencies with previous studies, such as the upregulation in patients with COVID-19 of ACE2 [22], FGF21 [41], CCL26 [54,55] and VWF [89,90] among others, suggest the potential validation of these novel findings. Further multi-center studies with larger cohorts including patients from different populations and ethnicities could lead to the development of novel biomarkers for the diagnosis and prognosis of COVID-19. Our data provide a valuable resource for the further functional characterization of novel players in a SARS-CoV-2 infection with in vitro and in vivo models to decipher their role in COVID-19 pathogenesis and their potential as therapeutic targets.

## 4. Materials and Methods

### 4.1. Study Cohort

The study involved 28 SARS-CoV-2 virus-infected patients (21 females and 7 males, age range 19–79) collected between April 2020 and November 2020. Nineteen of them were found to have other diseases, whereas the remaining nine were comorbidity-free. The control group consisted of 28 SARS-CoV-2 virus-negative subjects without any diseases (the so-called healthy controls) and additional 20 SARS-CoV-2 virus-negative subjects with additional diseases (the so-called disease control). The 19 patients with SARS-CoV-2 with additional diseases were age-and-sex-matched with 19 healthy controls as well as with 20 disease controls, who were age-and-sex matched and suffered from the same major disease(s) as the corresponding SARS-CoV-2 patient. The remaining nine SARS-CoV-2 virus-infected patients were age-and-sex-matched to the remaining nine healthy controls only. For one COVID-19 patient with chronic diseases, no age-and-sex-matched disease control was found, whereas two others needed to have two age-and-sex-matched disease controls owing to their clinical conditions (see Table 1 and Table S7 for details). Plasma samples of all subjects (patients and controls) together with respective clinical information were obtained from the Finnish Clinical Biobank, Tampere. All the participants provided informed consent. The study was conducted in accordance with the Declaration of Helsinki and was approved by the HUS ethics committee.

### 4.2. Proximity Extension Assay

The plasma samples were pipetted into a 96-well plate in a randomized order to circumvent the effects of experimental variables. The last column of the plate was left empty for two Olink samples, three negative and three inter-plate controls, which are used to calculate the intra-assay coefficient of variance, monitor background noise for the limit of detection calculation and compensate for potential variation between the runs, respectively. The sample plate was dispatched to Olink Proteomics (Uppsala, Sweden).

The PEA chemistry is based on antibody–antigen complexes. Two antibodies carrying unique and complementary DNA sequences bind to one specific protein in two different epitopes and the DNA tags hybridize and are amplified to generate a library of DNA fragments. The Olink Explore collection is sequenced by next-generation sequencing (NGS) which identifies each protein from a different sample using adaptors and unique barcode sequences [22]. First, the assay counts of a sample were divided with the extension control for that sample and block. A log2 transformation was applied, and the final step was subtracting the median of the plate controls. Then, the protein concentration can be translated using normalized protein expression (NPX) values. The higher NPX value corresponds to higher protein concentrations and vice versa. The sensitivity of PEA is comparable to or better than ELISA assays, down to fg/mL, and 99.8% of the assays exhibited no cross-reactivity due to the dual antibody recognition of the target protein combined with high-fidelity DNA hybridization and detection [92]. The Olink Explore collection consisting of 1472 proteins (1463 unique proteins) encompassing the cardiometabolic, neurology, inflammation, and oncology panels (369, 367, 368 and 368 proteins, respectively) was run for the extensive proteome profiling of the samples.

### 4.3. Statistical Analyses

Statistical analysis was performed in RStudio (version 1.3.1093) (RStudio, PBC, Boston, MA, USA) using R (version 4.0.3) (R Foundation for Statistical Computing, Vienna, Austria) [93,94]. First, the proteins and samples were filtered when the quality control was negative or the quantification led to values below the respective protein limit of detection (LOD) in at least 50% of the samples. The remaining NPX values were imputed with the respective LOD/√2 value. Between-group differences in protein expression levels were analyzed by means of the general linear model regression approach with analysis of contrasts using the “emmeans” R package (version 1.6.2.1). The whole regression modeling was divided into three main steps. In the first step, 19 SARS-CoV-2 virus-infected patients with comorbidities were involved to search for differential protein expression due to virus infection and/or coexistent comorbidities by comparing the protein expression levels in virus-infected patients to respective healthy and disease control subjects. The models used in this analysis comprised the presence of comorbidities as a nested confounding factor. In the second step of the analysis, the remaining nine SARS-CoV-2 virus-infected comorbidity-free patients were involved to search for DEPs due to virus infection itself in a comorbidity-free virus-infected group of patients by comparing the protein expression levels to those in the age-and-sex-matched healthy controls. No additional confounders were assumed in this analysis. In the last, third step of regression analysis, all 28 SARS-CoV-2 virus-positive patients were analyzed together to search for differential protein expression due to the time of sampling (early vs. late) and the presence/absence of antibodies, by simply comparing the respective groups. The models used in this step comprised the presence of comorbidities as a confounding factor, as well. All the general models did not include age and sex as confounding factors due to their balance between the compared groups according to the study design. The false discovery rate (FDR) in contrast analyses was controlled by means of the Benjamini and Hochberg correction [95]. Proteins were considered differentially expressed with an FDR-adjusted *p*-value < 0.05. KEGG and the gene ontology (GO) pathway enrichment analysis via active subnetworks from the STRING database was performed with the R package “pathfindR” (version 1.6.3) with FDR correction [96]. Graphics were generated with the R package “ggplot2” (version 3.3.5), except heatmaps that were generated with the R package “ComplexHeatmap” (version 2.6.2) and box and whisker plots that were generated with GraphPrism (version 9.3.1). The hierarchical clustering of differentially expressed proteins was performed after the transformation of the NPX values or means from each group of samples into z-score values using the Euclidean distances. The heatmap with means from each group of samples is split by k-means clustering. Protein networks from the STRING database were constructed in Cytoscape (version 3.8.2) with a confidence cut-off of 0.7.

### 4.4. Enzyme-Linked Immunosorbent Assay (ELISA)

ELISAs were carried out to detect serum IgM and IgG antibodies against the SARS-CoV-2 receptor-binding domain (RBD), a subunit of the spike S1 protein, and the nucleocapsid protein (NP). The assays were performed with kits from TestLine (Brno, Czech Republic) specific for IgM or IgG against the RBD (CoRM96 and CoRG96, respectively) or NP (CoNM96 and CoNG96, respectively). The IgG for both spike and NP together was detected by using a kit from Vircell Microbiologists (Granada, Spain, G1032). All of the assays were performed according to the manufacturer’s instructions.

## Figures and Tables

**Table 1:** Description of the clinical samples.

COVID-19 Patients	Sex	Age (Years)	Disease Control (DC)		Healthy Control	Sampling <3 Months of Infection	Antibodies *				
			DC1	DC2			IgG RBD	IgG NP	IgG (Spike + NP)	IgM RBD	IgM NP
Case 1	F	55			+		++	+	++	–	+
Case 2	M	78			+		++	+	+++	–	–
Case 3	F	41			+	+	–	+	+	+	–
Case 4	F	71	+	+	+		–	–	–	–	–
Case 5	F	36			+		–	–	–	–	–
Case 6	F	71	+		+		–	–	–	–	–
Case 7	F	54	+		+	+	–	–	–	–	–
Case 8	F	58	+		+		+++	++	+++	–	–
Case 10	F	53	+		+	+	–	–	–	–	++
Case 11	M	64			+	+	+++	+++	+++	+	+++
Case 12	F	61	+		+	+	–	–	+	–	–
Case 13	F	27	+		+	+	–	+	+	–	–
Case 14	F	45	+		+	+	+	+	+	+	–
Case 16	M	52	+	+	+		+	–	+	–	–
Case 17	M	64	+		+		–	–	–	–	–
Case 18	F	35			+		–	+	+	–	+
Case 19	F	31			+		–	–	–	–	–
Case 20	F	23	+		+		–	–	–	+	–
Case 21	F	53	+		+	+	+++	+++	+++	+	–
Case 22	F	30			+		–	–	+	–	–
Case 25	F	27			+	+	–	–	–	–	–
Case 26	M	62	+		+	+	–	–	–	–	–
Case 27	F	79	+		+	+	–	–	–	–	–
Case 28	F	36	+		+	+	–	–	–	–	–
Case 29	M	74	+		+	+	–	–	–	–	–
Case 30	F	19	+		+	+	–	–	–	–	–
Case 31	M	72	+		+		+	++	+++	–	+
Case 32	F	53			+		+	+	+++	–	–

* The SARS-CoV-2 antibody generation for different antigens in which the quantity of ‘+’ indicates the relative quantification for each antibody. Cases colored in blue indicate no comorbidities and those highlighted in yellow indicate no detectable antibodies. F, female; IgG, immunoglobulin G; IgM, immunoglobulin M; M, male; NP; nucleocapsid protein; RBD, receptor binding domain.

## Data Availability

The data presented in this study are available in the Supplementary Data.

## Supplementary Materials

The following supporting information can be downloaded at: https://www.mdpi.com/article/10.3390/ijms24076276/s1.
