# Supplementary material for: Plasma Proteomics Unveil Novel Immune Signatures and Biomarkers upon SARS-CoV-2 Infection
Source: Int J Mol Sci. 2023 Mar 27;24(7):6276. doi: 10.3390/ijms24076276 (PMC10093853; doi:10.3390/ijms24076276)
Supplement: Supplementary file 1 [file ijms-24-06276-s001.zip › ijms-2216868-supplementary/Supplementary figures.pdf]

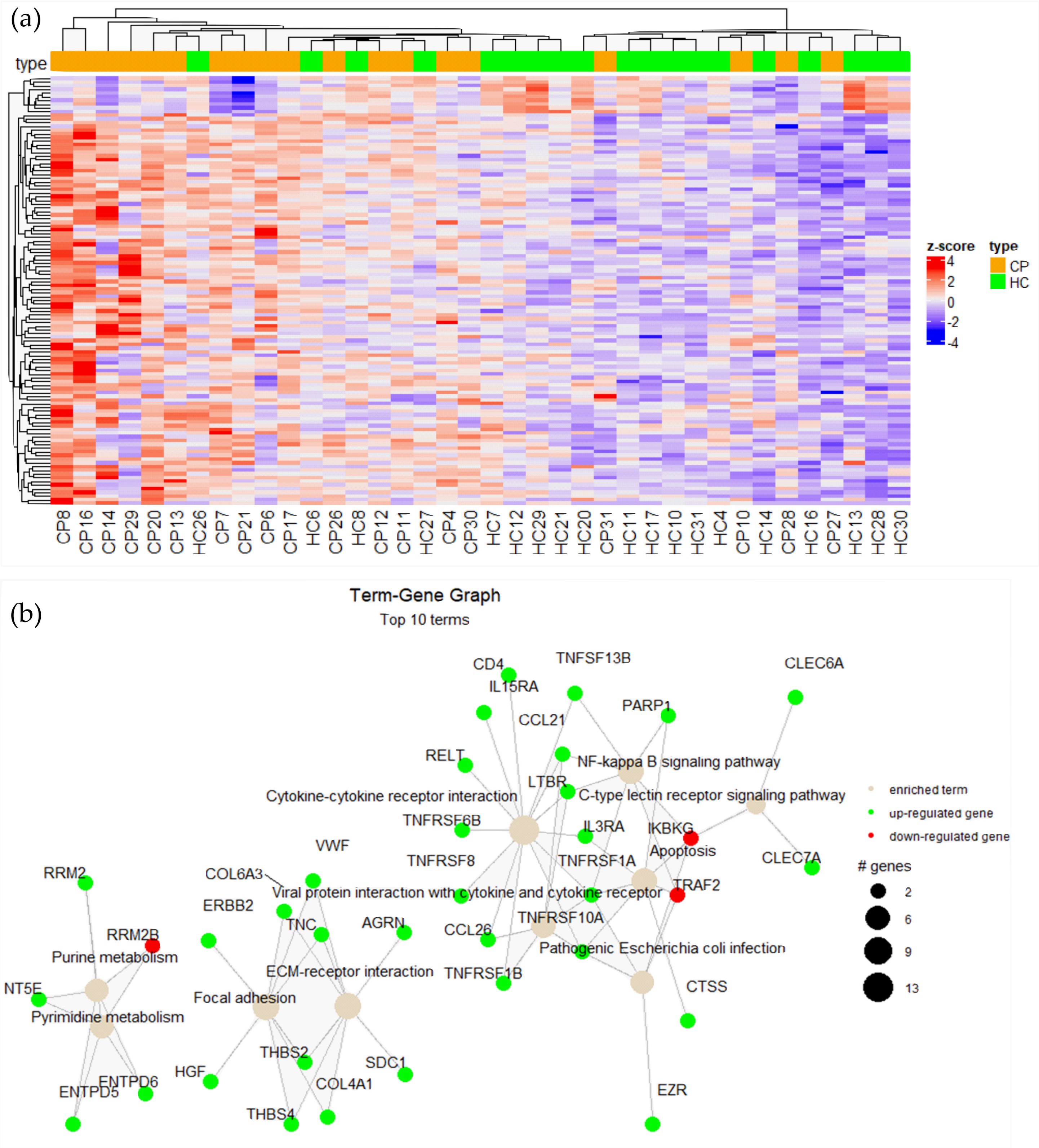

(a)
type
z-score
4
2
0
-2
-4
type
CP
HC
CP8
CP16
CP14
CP29
CP20
CP13
HC26
CP7
CP21
CP6
CP17
HC6
CP26
HC8
CP12
CP11
HC27
CP4
CP30
HC7
HC12
HC29
HC21
HC20
CP31
HC11
HC17
HC10
HC31
HC4
CP10
HC14
CP28
HC16
CP27
HC13
HC28
HC30
(b)
Term-Gene Graph
Top 10 terms
CD4
TNFSF13B
CLEC6A
IL15RA
PARP1
CCL21
RELT
NF-kappa B signaling pathway
LTBR
Cytokine-cytokine receptor interaction
C-type lectin receptor signaling pathway
TNFRSF6B
IL3RA
IKBKG
Apoptosis
VWF
TNFRSF8
TNFRSF1A
CLEC7A
RRM2
COL6A3
Viral protein interaction with cytokine and cytokine receptor
TRAF2
ERBB2
TNFRSF10A
TNC
AGRN
CCL26
RRM2B
Purine metabolism
Pathogenic Escherichia coli infection
ECM-receptor interaction
TNFRSF1B
NT5E
Focal adhesion
CTSS
Pyrimidine metabolism
THBS2
SDC1
HGF
ENTPD6
COL4A1
EZR
ENTPD5
THBS4
enriched term
up-regulated gene
down-regulated gene
genes
2
6
9
13

Figure S1: Protein expression in clinical groups and altered pathways in patients. (**a**) Heatmap of differentially expressed proteins (DEPs) between patients infected with SARS-CoV-2 with comorbidities and healthy controls, with z-score by row normalization and distributed by hierarchical clustering; (**b**) network of KEGG-enriched terms from DEPs between patients with comorbidities and healthy controls. Green (upregulated in patients) and red (downregulated in patients) dots indicate statistical DEPs. The node size indicates the number of genes involved in the term.

(a)

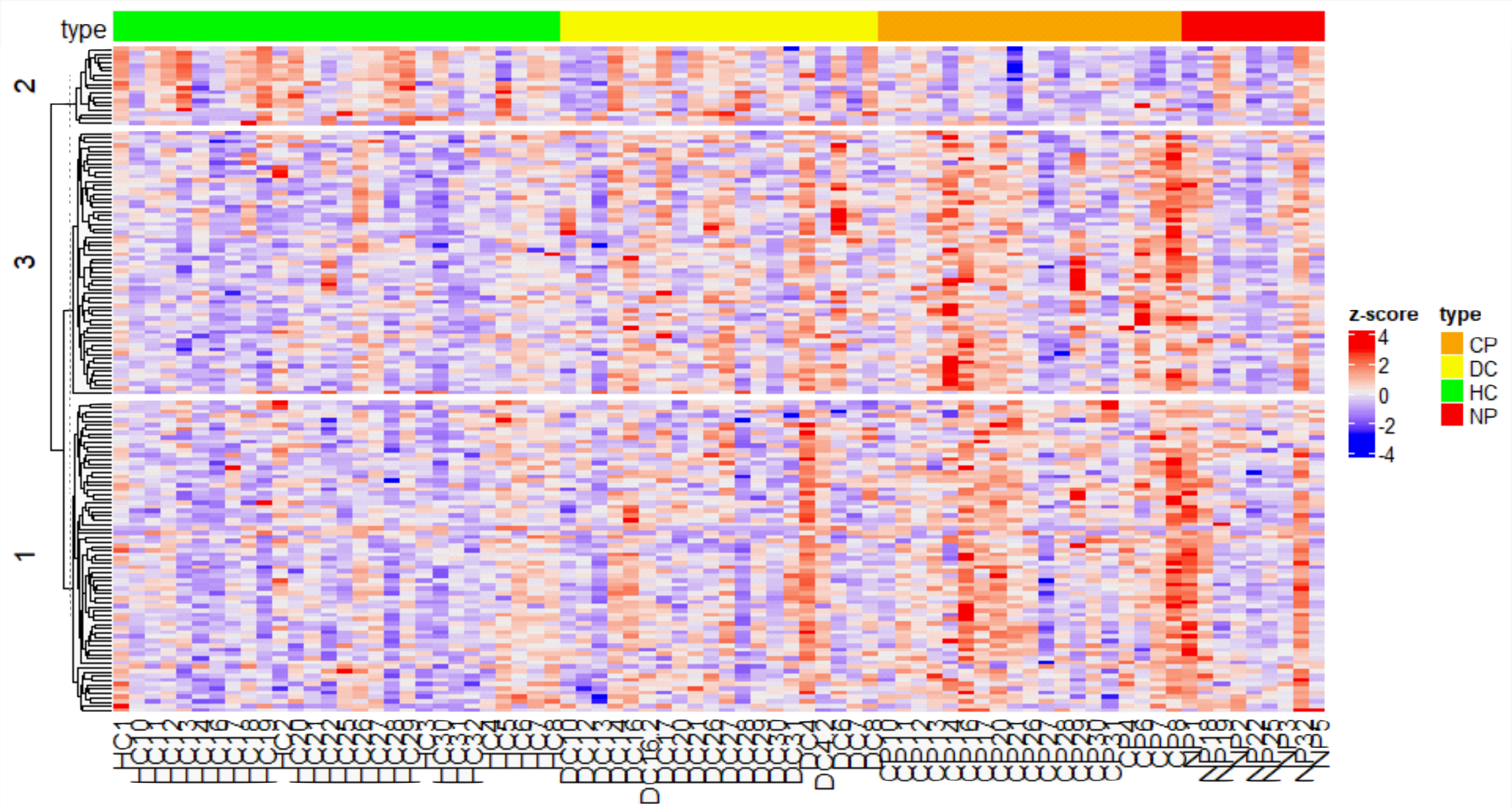

type
2
3
1
z-score
4
2
0
-2
-4
type
CP
DC
HC
NP

(b)

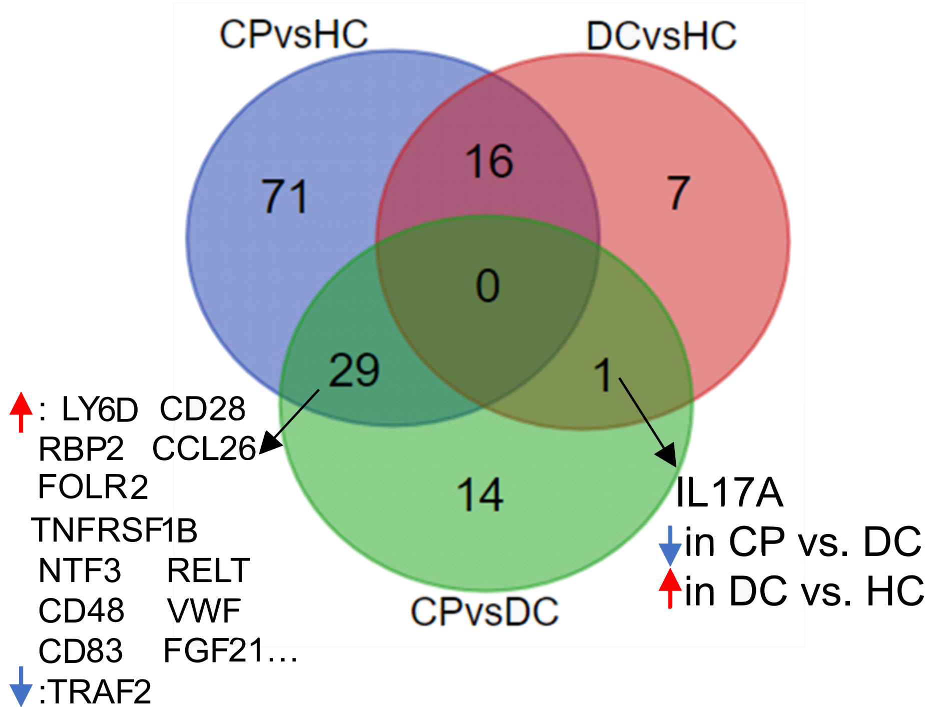

CPvsHC
DCvsHC
CPvsDC
71
16
7
0
29
1
14
: LY6D CD28
RBP2 CCL26
FOLR2
TNFRSF1B
NTF3 RELT
CD48 VWF
CD83 FGF21…
:TRAF2
IL17A
in CP vs. DC
in DC vs. HC

Figure S2: Protein expression in clinical groups and altered pathways in patients. (**a**) Heatmap of the DEPs among patients with comorbidities, patients without comorbidities, healthy controls, and disease controls with z-score by row normalization and distributed by hierarchical clustering. The 3 clusters were generated by K-means algorithm; (**b**) Venn diagram with DEPs of patients with comorbidities versus healthy controls, DEPs of disease controls versus healthy controls, and DEPs of patients with comorbidities versus disease controls. The red and blue arrows represent upregulation and downregulation, respectively.

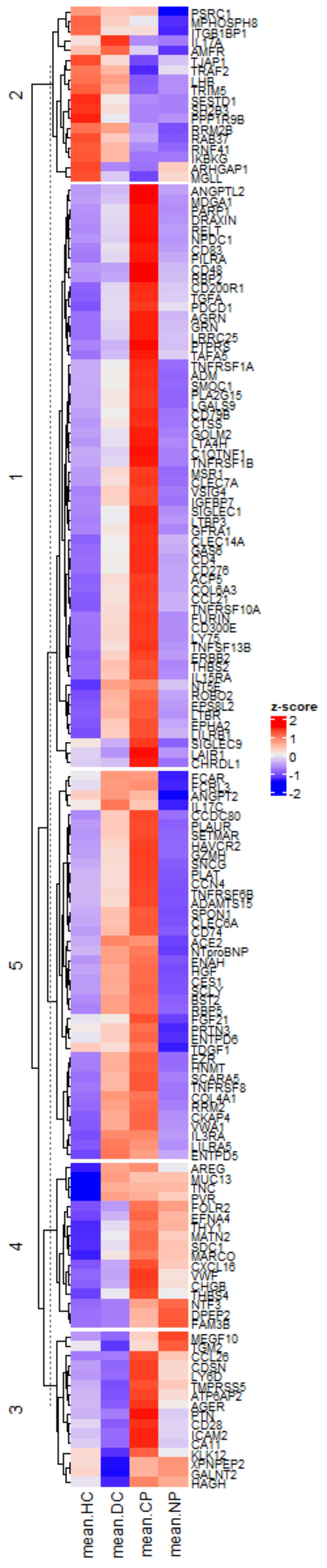
2
1
5
4
3
PSRC1
MPHOSPH8
ITGB1BP1
AMFR
TJAP1
TRAF2
LHB
TRIM5
SESTD1
PPP1R9B
RAB37
RNF41
IKBKG
ARHGAP1
MGLL
ANGPTL2
MDGA1
DRAXIN
NPDC1
CD83
PILRA
CD48
CD200R1
TGFA
PDCD1
AGRN
GRN
LRRC25
TAFA5
TNFRSF1A
ADM
SMOC1
PLA2G15
LGALS9
CTSS
GOLM2
LTA4H
C1QTNF1
TNFRSF1B
MSR1
CLEC7A
VSIG4
IGFBP7
SIGLEC1
LTBP3
GFRA1
CLEC14A
GAS6
CD4
CD276
COL6A3
CCL21
TNFRSF10A
FURIN
CD300E
LY75
TNFSF13B
ERBB2
THBS2
IL15RA
NT5E
ROBO2
EPS8L2
LTBR
EPHA2
LILRB1
SIGLEC9
LAIR1
CHRDL1
FCAR
FCRL3
ANGPT2
IL17C
CCDC80
PLAUR
SETMAR
HAVCR2
GZMH
SNCG
PLAT
CCN4
TNFRSF6B
ADAMTS15
SPON1
CLEC6A
CD74
ACE2
NTproBNP
ENAH
HGF
CES1
BST2
RBP5
FGF21
PRTN3
ENTPD6
TDGF1
EZR
HNMT
SCARA5
TNFRSF8
COL4A1
RRM2
CKAP4
VWA1
IL3RA
LILRA5
ENTPD5
AREG
MUC13
TNC
PVR
FOLR2
EFNA4
MATN2
SDC1
MARCO
CXCL16
VWF
CHGB
NTF3
DPEP2
FAM3B
MEGF10
CCL26
CDSN
LY6D
TMPRSS5
ATP6AP2
AGER
CD28
ICAM2
CA11
KLK12
XPNPEP2
GALNT2
HAGH
z-score
2
1
0
-1
-2
mean.HC
mean.DC
mean.CP
mean.NP

Figure S3: Clusters of protein expression in clinical groups by mean. Heatmap of the mean of DEPs for each clinical group among patients with comorbidities, patients without comorbidities, healthy controls, and diseases controls with z-score by row normalization and distributed by hierarchical clustering. The 5 clusters were generated by K-means algorithm.

RNF41

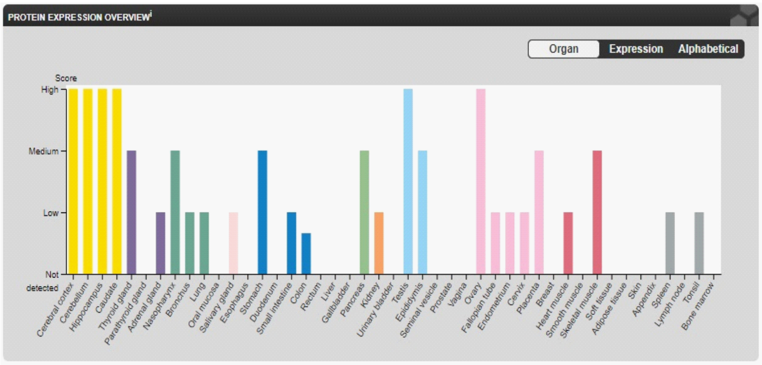

SPON2

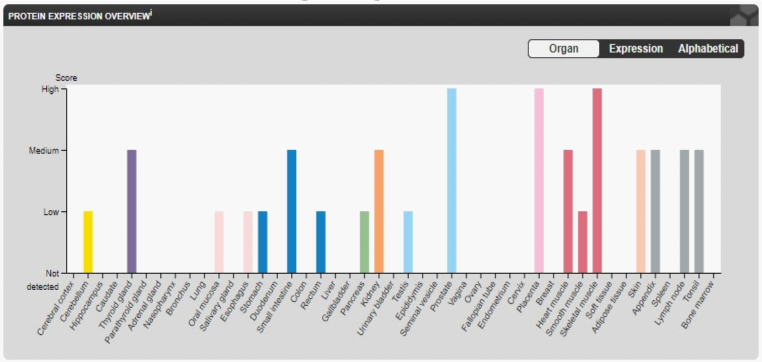

HGF

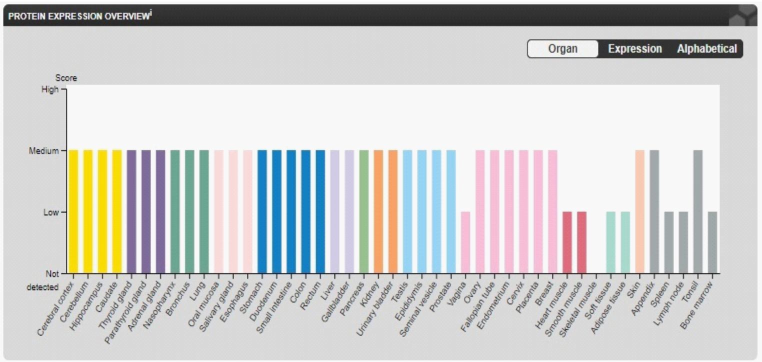

PTN

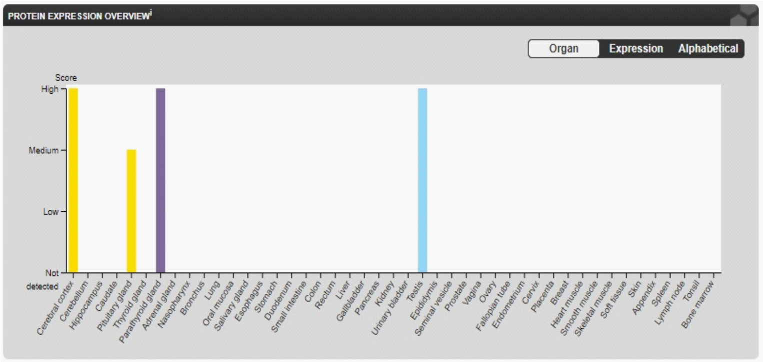

LRRC25

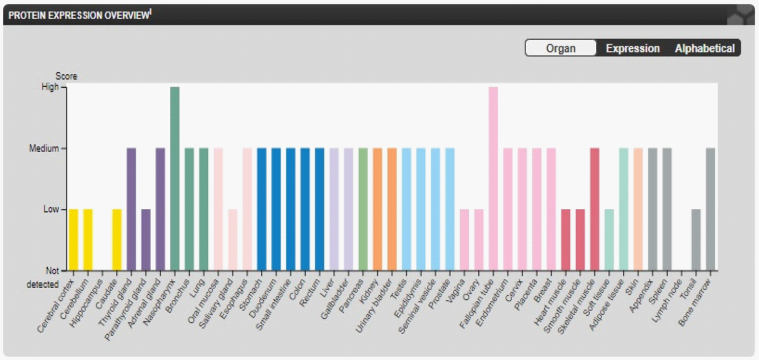

RBP2

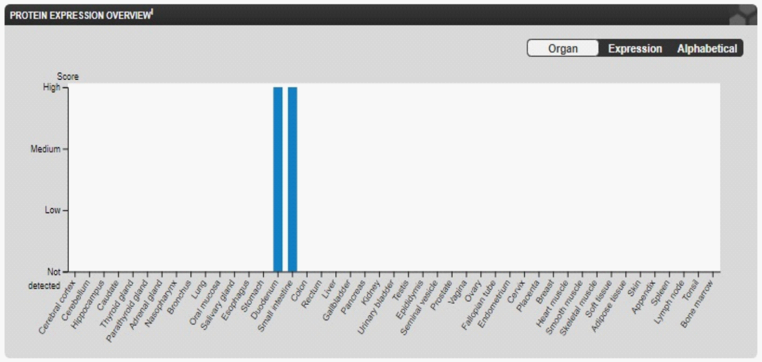

THY1

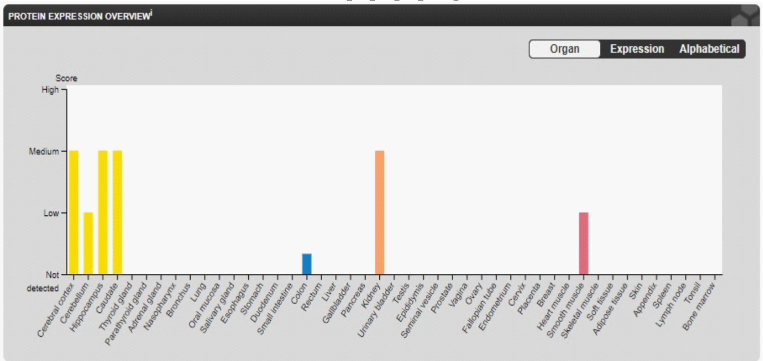

SMOC1

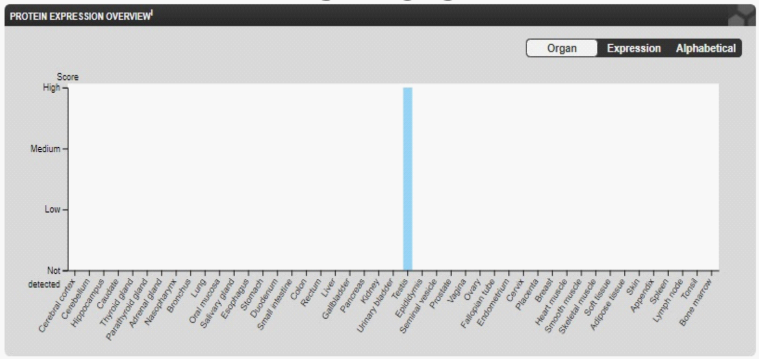

NPDC1

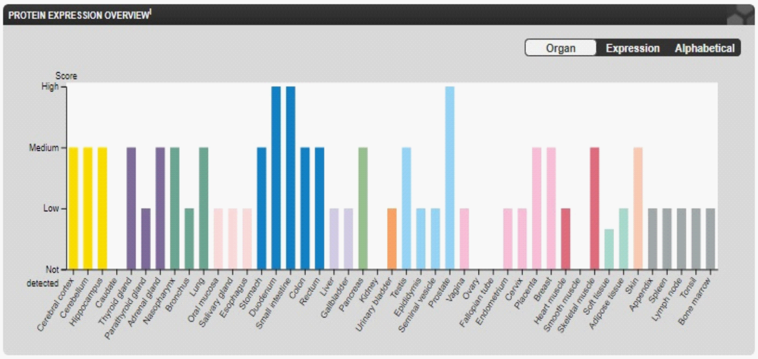

GOLM2

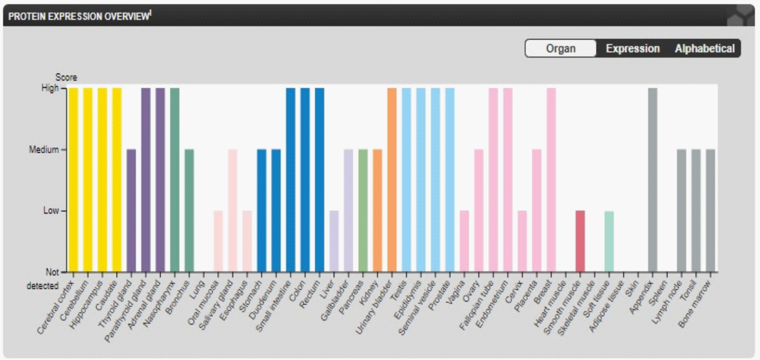

Figure S4: Cell-specific expression of selected proteins from the Human Protein Atlas. Bar plot with relative quantification (score) for each protein in different cell types.
